# Climate Change Impacts on the Tree of Life: Changes in Phylogenetic Diversity Illustrated for *Acropora* Corals

**DOI:** 10.3390/biology1030906

**Published:** 2012-12-14

**Authors:** Daniel P. Faith, Zoe T. Richards

**Affiliations:** 1 The Australian Museum, Sydney, NSW 2010, Australia; 2 Western Australian Museum, Welshpool, WA 6106, Australia; E-Mail: zoe.richards@museum.wa.gov.au

**Keywords:** biodiversity, phylogeny, PD, risk analysis, evosystem services, extinction, tipping points, biotic homogenization, corals, *Acropora*

## Abstract

The possible loss of whole branches from the tree of life is a dramatic, but under-studied, biological implication of climate change. The tree of life represents an evolutionary heritage providing both present and future benefits to humanity, often in unanticipated ways. Losses in this evolutionary (evo) life-support system represent losses in “evosystem” services, and are quantified using the phylogenetic diversity (PD) measure. High species-level biodiversity losses may or may not correspond to high PD losses. If climate change impacts are clumped on the phylogeny, then loss of deeper phylogenetic branches can mean disproportionately large PD loss for a given degree of species loss. Over time, successive species extinctions within a clade each may imply only a moderate loss of PD, until the last species within that clade goes extinct, and PD drops precipitously. Emerging methods of “phylogenetic risk analysis” address such phylogenetic tipping points by adjusting conservation priorities to better reflect risk of such worst-case losses. We have further developed and explored this approach for one of the most threatened taxonomic groups, corals. Based on a phylogenetic tree for the corals genus *Acropora*, we identify cases where worst-case PD losses may be avoided by designing risk-averse conservation priorities. We also propose spatial heterogeneity measures changes to assess possible changes in the geographic distribution of corals PD.

## 1. Introduction

Human-induced global climate change has been implicated as one important driver in the ongoing global biodiversity crisis [1,2,3,4,5,6,7]. Such studies have documented climate change impacts across the various levels of biological variation that are conventionally equated with “biodiversity”, including genes, species, and ecosystems. Our paper focuses on the impacts of climate change on another important level of variation—phylogenetic diversity [8,9]. Loss of phylogenetic diversity arises from the loss, through species extinctions, of evolutionary branches from the tree of life (phylogeny). Based on a phylogenetic diversity measure, PD (defined below), phylogenetic pattern can be used to assess expected loss of biodiversity at the level of features or attributes of species. Our first goal in this paper is to briefly review findings from the relatively small number of studies that have examined climate change impacts on phylogenetic diversity. We then will focus in more detail on one of those key taxonomic groups of great interest—the reef-building scleractinian corals. Based on an inferred phylogenetic pattern for the genus *Acropora*, we will develop and illustrate some useful phylogenetic diversity indices for quantifying various aspects of climate-change impacts on phylogenetic diversity. 

Our paper is a contribution to this special issue of *Biology*, with a thematic focus on the impacts of human-induced climate change on “global biological systems” or “life support systems of the planet”. The issue’s overview [10] highlights typical systems-level topics, including interactions, thresholds, cascading effects, and loss of functions. Our focus on phylogenetic diversity at first may not seem to fit well into this big-system perspective. The typical rationale for Earth system analyses, integrating humans and environment, is that analyses of the whole system provide a better understanding of the system components as well as the whole [11,12]. In contrast, our phylogenetic pattern approach, in focusing on attributes or features, may appear closer to a “reductionist” focus on the smaller components, not the whole system. However, we will argue that our approach in fact contributes to any complete “systems” approach that is truly integrative and links broadly to human well-being issues. 

We develop our argument by first considering recent characterizations of biodiversity in terms of functions and processes. Earth system science typically focuses on the problem of maintaining functioning systems. For example, Australia’s vision statement for Earth system science [13] sees the “overarching issue” as how to secure “a well-functioning and resilient earth system for the indefinite future.” This focus on functioning systems sometimes has meant that “biodiversity” has been re‑defined as primarily about functions and processes, giving less emphasis to the attributes and patterns conventionally used to characterize living variation. For example, a recent proposal for a systemic (“systems thinking”) framework for biodiversity and its conservation [11] defined biodiversity as “all life on earth across all levels (genes, population, and species—including humans, assemblages, ecosystems/landscapes, and the ecosphere) and the ecological, cultural, and evolutionary processes that sustain it.” Beyond the vague reference to “all life”, biodiversity here is characterized in terms of processes. Thus, the seemingly obvious idea that “biodiversity” should be about “diversity”—extent of variation [14]—is absent.

This processes perspective is echoed in the new international initiative, “Future Earth” [15], on “Earth system research”: “Biodiversity is a key to development, in that it provides the basis for fully functioning ecosystems, which are important for human well-being and economies, and the loss of biodiversity has been shown to undermine development”. This Future Earth framework also refers to the challenge of “identifying the linkages to earth system functions and processes including biodiversity”. Again, biodiversity is characterized here through function and process, and in this way is seen as under-pinning “development” and human well-being [16]. Apparent support for this perspective is found in the argument [11] that a process-based definition of biodiversity is needed because a traditional focus on attributes and patterns ignores humans: “Historically, views and measurements of biodiversity often focused on characterizing the attributes of observable patterns… and afforded less attention to processes. Often, these views of biodiversity see humans as separate from the rest of nature…”

These arguments might seem to imply that an attributes/pattern approach, such as phylogenetic diversity, does not fit into a systems perspective. However, the reality is that attributes and pattern not only provide the basic elements used for quantifying variation and therefore “biodiversity”, but also provide a fundamental link to human well-being. This link to human well-being is apparent in the historical rationale for the conservation of biodiversity (living variation): the need to preserve “option values” [14,17,18,19,20]. Option values of biodiversity reflect the possibility of future, often unanticipated, human uses and benefits [8,14,17,18,19,20]. Thus, the relationship between biodiversity and human well‑being extends well beyond the narrow idea of “biodiversity” as supporting functioning systems. This relationship also extends beyond the observation (noted above) that “the loss of biodiversity has been shown to undermine development” to include the obvious point that loss of biodiversity sometimes could *result from* development, and so require trade-offs. Consequently, biodiversity is not just a cog in the wheel of a functioning Earth system in support of human development. Human well‑being from biodiversity, particularly relating to benefits for future generations, may involve both trade-offs and synergies with other needs of society. This contrasts with a narrow “systems science” perspective [16] in which biodiversity and society “are not separate domains to be traded off against one another”. These themes are clear when we consider biodiversity measures based on phylogenetic pattern and phylogenetic diversity. The tree of life represents an evolutionary heritage providing both present and future benefits to humanity, often in unanticipated ways. Returning to the themes for this special issue, we can say that losses in this evolutionary “life-support system” represent losses in current and future benefits for humans [18,19,20]. Thus, both attributes and pattern are central to Earth system science. 

We therefore view phylogenetic diversity as contributing to an inclusive systems science in two ways. First, phylogenetic pattern is a good proxy for the “attributes” that result from evolutionary processes [8,9], and so is part of a “life support system” that provides both current and possible future benefits from such attributes (“evosystem” services; [18]). Second, phylogenetic diversity is part of a larger systems approach to sustainability that examines global environmental change and decision‑making, and investigates how we can balance different needs of society [21,22]. An expanded systems approach requires practical measures and indices of biodiversity. Here we establish measures and indices reflecting the option values associated with phylogenetic diversity. We choose to apply the new indices to hard corals because this group is highly threatened, with one-third of existing species falling into an elevated category of threat under IUCN criteria [23]. Furthermore, the keystone functional roles of reef-building corals such as *Acropora* play in coral reef ecosystems are well established [24]. In addressing “system responses to climate change”, it has been reported that “There are already indications of dramatic impacts of global warming at the system level, particularly in the arctic and for coral reef systems. Mass coral bleaching driven by warmer sea temperatures has killed vast numbers of corals across the tropics, causing some reefs to lose their ecosystem structure and functions.” [25]. We see the quantification coral biodiversity option values as complementing these studies on ecosystem structure and function. 

To develop these arguments, we will first introduce the phylogenetic diversity measure, PD [8,26] and review studies examining climate change impacts on PD. We then develop the phylogenetic pattern for *Acropora* species using a new molecular phylogeny and use this framework to explore a range of indices based on PD that capture various aspects of change.

## 2. The Phylogenetic Diversity Measure, PD

**Figure 1:**
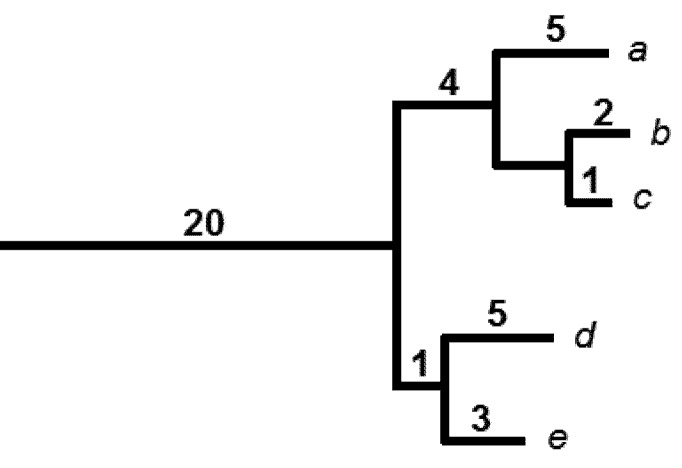
A hypothetical phylogenetic tree for species *a* through *e*. Branch lengths are shown above branches. The PD is 41 for this set of species (20 + 5 + 4 + 2 + 1 + 5 + 1 + 3). If species *a* was lost, 5 units of PD would be lost. Successive losses of species would imply PD losses of similar magnitude. However, the loss of the last species of the clade would imply that the deeper branch of 20 units is now lost as well.

The PD measure is based on the assumption that shared ancestry for two species indicates that they have shared attributes or features. The PD of a subset of species from the phylogenetic tree is calculated as the minimum total length of all the phylogenetic branches required to connect all those species on the tree. PD provides a natural way to talk about future uses and benefits provided by species—the option values of biodiversity. PD’s evolutionary process model, where shared ancestry accounts for shared features, means that PD can be interpreted as counting-up the features represented by a given set of species (Figure 1); any subset of species that has greater PD will be expected to have greater feature diversity. In this way, PD values indicate option values at the level of features of species [8,26]. 

A study by Forest *et al.* [27] nicely demonstrated how PD captures option values. They analyzed a large plant phylogeny including taxa with a variety of known human uses (medicinal, food, *etc.*). For their analyses, they assumed that we did not yet know about these uses, and showed how conserving species to maximize total PD (say, for given budget) was best way to maximise the chance of preserving a wide range of uses.

We can interpret various PD calculations as if they were counting-up features. For example, the loss of a species from a protected set is interpreted as a loss in the total number of features represented by the set (Figure 1). A family of PD calculations extends conventional species-level indices to the features level. For example, “PD-endemism” indicates the extent to which evolutionary features are restricted to a given region [28,29]. 

The PD measure has provided a phylogenetic basis for setting conservation priorities among species or areas [8,9,27], and is regarded “as a leading measure in quantifying the biodiversity of a collection of species” [30] and as the phylodiversity metric of choice in conservation research” [31]. PD has also been characterized as a “unique and important measure of biological diversity” [32], and as “a resonant symbol of the current biodiversity crisis” [33]. 

A larger number of species in a set generally implies a larger PD of the set [31,34,35]. Based on this general relationship, it is sometimes argued that conservation priorities based on maximizing species richness will also conserve phylogenetic diversity [36], but see [27]. Faith and Williams [37] suggested that the PD—species relationship could be approximated by a power law curve, and this proposal has gained support from the empirical work of [31]. This relationship has interesting implications for the expected magnitude of PD loss for any given amount of species loss. The shape of the curve implies that initial losses of species, from climate change or other impacts, will mean only small losses in PD, while later species losses can mean steeper declines in PD.

An important consideration is that this power curve relationship depends on “random” species losses from the tree. In reality, the amount of actual PD loss depends on whether the species extinctions are clumped or well-dispersed on the phylogenetic tree (for review and discussion, see [35,38]). The decoupling of species loss and PD loss has recently been documented [39]. This PD study demonstrates that “phylogenetic diversity and species richness are decoupled at small and medium scales and are imperfectly associated at large scales”, and concludes that the results are “consistent with expected early effects of climate change”.

Faith [18] referred to possible scenarios where phylogenetically clumped impacts, spread out over time, can mean that initial species losses produce small incremental PD losses, until all descendent species from a longer branch are lost and the PD falls precipitously (Figure 1). They outlined a form of phylogenetic risk analysis to guide conservation decisions that try to reduce risk of these worst case losses, or “tipping point” outcomes. We return to this problem below. 

## 3. A Brief Review of Climate Change Impacts on PD

The PD-species power curve relationship suggests that climate change impacts initially (for a small number of affected species) might imply small PD loss. However, we have noted that the actual extent of PD loss depends on whether species extinctions are clumped or well-dispersed on the phylogenetic tree. A number of studies investigating climate change impacts have found relatively small PD losses. For example, climate change impacts that are spread out over a phylogenetic tree effectively ensure that most deep branches throughout the tree have at least one surviving descendent [40]. Another study found small PD loss [38] given species losses were phylogenetically dispersed among plant, bird and mammal taxonomic groups over continental Europe.

In contrast, some studies have found that species extinction is concentrated on the phylogeny (“clumped”), resulting in a disproportionate loss of PD. One cause of such disproportionate loss is the occurrence of entire clades in the same threatened location or region [41]. For example, this kind of phylogenetic clumping for mammals accounts for the finding that several biodiversity hotspots in southern Asia and Amazonia are likely to lose “an unexpectedly large proportion of PD” [42]. 

Disproportionate PD loss also may arise when entire clades share the same key trait(s) implying vulnerability to climate change. For example, Willis *et al.* [43] used a time series of abundance of flowering plants to indicate possible human-caused impacts. This study of plants revealed that reductions in abundance were not randomly distributed across the plant phylogeny, with some plant families having an over-representation of declining species [43]. This phylogenetic clumping was attributed to the fact that these families could be characterized as having flowering times that do not closely track temperature—implying greater vulnerability to climate change. However, sometimes clumped impacts may reflect a combination of traits and geography. Baillie *et al.* [44] examined red list species (reflecting climate change and other threats) and found that “a number of families have significantly more threatened species than would be expected on average, while others have far less…entire evolutionary lineages are likely to go extinct very quickly.” 

Climate change impacts may be well-dispersed over a given phylogenetic tree, but reveal hotspots of clumped impacts at a finer phylogenetic resolution. For example, based on an existing large phylogenetic tree for corals, Faith *et al.* [18] observed that, while threatened species appear well‑dispersed on the overall phylogenetic tree, sometimes entire monophyletic groups (existing families and genera) within the tree fall into IUCN threatened (or near-threatened) classes. These included all species within the genera *Catalaphyllia*, *Physogyra*, and *Euphyllia*. Faith *et al.* suggested that these may represent phylogenetic tipping points given that each group represents the only descendent taxa of a relatively long phylogenetic branch (see Figure 1). Furthermore, an important preliminary assessment of PD loss for corals [45] suggests that there is no significant phylogenetic clumping of extinction risk. On the other hand, this study reports that key traits that possibly relate to vulnerability were typically shared by close relatives.

These studies raise challenges for the development of PD-based indices that reflect changes in phylogenetic diversity. The overall impacts from climate change may be phylogenetically dispersed, and overall PD loss consequently small, but certain parts of the phylogenetic tree nevertheless may show clumped impacts. This is made more complicated by the fact that “clumped” impacts can be measured in a number of ways, and measures applied to the overall tree may not reflect patterns for sub-trees [35]. The corals studies referred to above (and others) suggest that the conventional focus on the complete phylogeny of a group (where the conclusion may be that there is relatively small PD losses) may conceal the impacts at the finer phylogenetic scale. Further, studies so far suggest that loss of PD is not the only impact of interest. For example, net losses of PD can be small but nevertheless, there can be dramatic changes in the geographic distribution of PD [38]. In the sections below, we describe our study context and then explore PD indices to address these issues.

## 4. Phylogeny of *Acropora* — Rationale and Methods

### 4.1. Introduction to Acropora as a Model Group for Extinction Risk Studies

The genus *Acropora* (commonly known as staghorn corals) is an ideal model group for studies of extinction risk because 50% of *Acropora* species are predicted to face an elevated risk of extinction this century under IUCN categories and criteria [23]. Species in this genus display complex spatio‑temporal patterns and an extensive literature exists on their global ranges [46,47,48,49]. Many species of *Acropora* are described as rare, occurring in small, restricted, isolated and/or disjunct populations [47,49,50]. In general, species of *Acropora* are extremely susceptible to coral bleaching [51], changes in water quality, disease [52,53] and predation (e.g., the corallivorous starfish *Acanthaster planci*; and the gastropods *Coralliophila abbreviata* and *Drupella* spp.). 

*Acropora* is the largest extant genus of reef-building corals. The genus *Acropora* was formally known as *Madrepora* (Linnaeus 1758) before the name *Acropora* was introduced by Oken in 1815 and reintroduced by Verrill in 1901. In 1999, the first full monograph of the genus was published since Brook (1893) [47]. Under this system, 114 species were described. Although another 250+ species of *Acropora* have been described—some are represented by fossil material only, and others are *nomina nuda*, meaning they are not officially validated (*i.e.*, some of the species described in [49]); and many others were placed into synonymy after extensive examination of type material [47]. However, 14 of the new *Acropora* species described in [49] have recently been validated [54,55]. In addition, 6 new species have been described since the 1999) revision [54,56,57,58,59].

Currently, the morphological phylogeny portrays the rudis group to be the oldest living lineage and the echinata group to be the youngest [47]. However some fundamental differences are apparent with the published molecular phylogenies. For example, mitochondrial DNA suggests the Atlantic species—*A. cervicornis* and *A. palmata* are the basal lineage [60,61] rather than members of the rudis group, *A. longicyathus* occurs in a near basal position in the molecular phylogeny [60] despite belonging to the ‘most recently evolved’ echinata group [47]. Most *Acropora* phylogenies have included only common and widespread species, however the inclusion of rare species in a new phylogeny [62], suggest that not all rare species are recently evolved and hence, there is substantial risk that novel phylogenetic information will be lost if rare and threatened species are driven to extinction by the end of this century as predicted [23].

For this study, the threatened status of 173 coral species was downloaded from the IUCN Red List ([63], see Table 1 and Table 2). For this assessment, nearly all extinction risk assessments were made with the IUCN criterion that uses measures of population reduction over time [23]. Most reef building corals do not have sufficient long-term species-specific monitoring data to calculate actual population trends; thus, to complete the global coral threatened species assessments, a less quantitative (surrogate) approach was adopted to assess the status of coral species. Specifically, the vast majority of coral species were assessed under a criterion in which population reductions are estimated from the “decline in habitat quality.” They were based therefore on a conservative interpretation of the most current global and regional estimates of coral reef status in 17 regions across the world [64]. For each species, a weighted average was calculated by multiplying the area of reef within the species distribution by the percent of total coral cover loss or the combined percent of total coral cover loss and critically declining reef [64]. Overall, estimates of habitat loss (in conjunction with life history traits and susceptibility) are used as a surrogate for population reduction, assuming the generation time of corals is 10 years [23]. Therefore, rates of population decline for each species have their basis in the rate of habitat loss within its range adjusted by an assessment of the species-specific response to habitat loss (*i.e.*, more-resilient species have slower rates of decline).

In the next sections, our calculations using estimated extinction probabilities will follow the “pessimistic case” [65] to convert these categories to estimated extinction probabilities (Table 1).

**Table 1:** Number of species in the different IUCN categories of threat according to [23]. Conversion to extinction probability follows [65].

IUCN Category	Number of *Acropora* species (n = 173)	Percentage of *Acropora* species	Conversion to extinction probability
Critically Endangered (CE)	2	1.2%	0.99
Endangered (E)	3	1.7%	0.9
Vulnerable (V)	49	28.3%	0.8
Near Threatened (NT)	22	12.7%	0.4
Least Concern (LC)	27	15.6%	0.2
Data Deficient (DD)	64	37%	-
Not Assessed	6	3.5%	-

### 4.2. Phylogenetically-Informative Markers

Various markers are available to assess phylogenetic relationships within the *Acropora*, including ribosomal DNA (rDNA) and ITS (internal transcribed spacer) sequences [66,67,68]. However, rDNA performs suboptimally when testing evolutionary relationships in the *Acropora* because it is a fast evolving genus [69], and extremely high rDNA diversity can predate species divergence [70]. Single‑copy nuclear markers have also been used in *Acropora* phylogenetics (e.g., Mini-C [70,71], Cnox2 [72]; Calmodulin [73]); and an extensive published datasets exists for the Pax-C 46/47 nuclear intron [60,62]. However extensive intra-individual polymorphisms are observed when this marker is cloned (*i.e.*, when different alleles are sequenced from a single PCR product from a single individual, the different alleles can occur in divergent clades), and such genetic heterogeneity greatly complicates the interpretation of extinction risk.

For the purposes of this study we utilize a single-copy mitochondrial marker, the putative mitochondrial control region rns-cox3, for which an extensive amount of data is publicly available (via Genbank, see Table 2). Being maternally inherited, there is one haploid per genome unlike repetitive markers (such as rDNA) that occur in multiple copies. Further, single-copy mitochondrial introns are expected to accumulate mutations relatively rapidly providing many potentially phylogenetically informative characters; lineage sorting is expected to occur relatively rapidly which further benefits the interpretation of evolutionary relationships [74].

### 4.3. Molecular Phylogenetic Analysis by Maximum Likelihood Method

Mitochondrial rns-cox3 data with sufficient coverage was available for 65 species and these were downloaded from Genbank [75] (See Table 2 for species names and source details). A total of 640 positions were included in the final dataset and 1st + 2nd + 3rd coding positions + non-coding positions were included. All positions with less than 95% site coverage were eliminated. That is, fewer than 5% alignment gaps, missing data, and ambiguous bases were allowed at any position. For the purposes of this study, evolutionary analyses were conducted in MEGA5 [76].

**Table 2:** Genbank Accession numbers and details of the 65 species included in the *Acropora* phylogeny.

Species (Authority)	GENBANK Accession No.	Source	Species Group in Wallace 1999	IUCN status
*Acropora abrotanoides* (Lamarck, 1816)	FJ899076.1	[77]	robusta group	LC
*Acropora aculeus* (Dana, 1846)	aculeus2_39IF	[78]	latistella group	V
*Acropora acuminata* (Verrill, 1864)	acuminata2_13IF	[78]	muricata group	V
*Acropora aspera* (Dana, 1846)	EU918267	[62]	aspera group	V
*Acropora austera* (Dana, 1846)	EU918228.1	[62]	rudis group	NT
*Acropora batunai* (Wallace, 1997)	EU918250.1	[62]	echinata group	V
*Acropora carduus* (Dana, 1846)	carduus1_10IF	[78]	echinata group	NT
*Acropora caroliniana* (Nemenzo, 1976)	EU918264.1	[62]	loripes group	V
*Acropora cerealis* (Dana, 1846)	EU918248.1	[62]	nasuta group	LC
*Acropora cervicornis* (Lamarck, 1816)	GQ863996	[79]	cervicornis group	CE
*Acropora chesterfieldensis* (Veron and Wallace, 1984)	EU918279.1	[62]	loripes group	LC
*Acropora cytherea* (Dana, 1846)	cythereaD7hetplasIF	[78]	hyacinthus group	LC
*Acropora derewanensis* (Wallace, 1997)	EU918263.1	[62]	horrida group	V
*Acropora digitifera* (Dana, 1846)	EF206519	[80]	humilis group	NT
*Acropora divaricata* (Dana, 1846)	AY026432	[60]	divaricata group	NT
*Acropora donei* (Veron and Wallace, 1984)	donei1_121IF	[78]	selago group	V
*Acropora elseyi* (Brook, 1892)	elseyi2_22IF	[78]	echinata group	LC
*Acropora florida* (Dana, 1846)	AY026435.1	[60]	florida group	NT
*Acropora muricata*(Dana, 1846)	AY026436.1	[60]	muricata group	NT
*Acropora gemmifera* (Brook, 1892)	EU918256.1	[62]	humilis group	LC
*Acropora globiceps* (Dana, 1846)	EF206434.1	[80]	humilis group	V
*Acropora granulosa* (Milne Edwards and Haime, 1860)	EU918286	[62]	loripes group	NT
*Acropora horrida* (Dana, 1846)	horridaIF	[78]	horrida group	V
*Acropora humilis* (Dana, 1846)	EU918282.1	[62]	humilis group	NT
*Acropora hyacinthus* (Dana, 1846)	AB361095.1	[81]	hyacinthus group	NT
*Acropora jacquelineae* (Wallacew, 1994)	EU918284.1	[62]	loripes group	V
*Acropora kimbeensis* (Wallace, 1999)	EU918268.1	[62]	nasuta group	V
*Acropora kirstyae* (Veron and Wallace, 1984)	EU918215.1	[62]	horrida group	V
*Acropora latistella* (Brook, 1891)	AY026443.1	[60]	latistella group	LC
*Acropora loisetteae* (Wallace, 1994)	EU918273.1	[62]	selago group	V
*Acropora lokani* (Wallace, 1994)	EU918270.1	[62]	loripes group	V
*Acropora longicyathus* (Milne Edwards and Haime, 1860)	EU918221.1	[62]	echinata group	LC
*Acropora loripes* (Brook, 1892)	EU918205.1	[62]	loripes group	NT
*Acropora microphthalma* (Verrill, 1859)	EU918203.1	[80]	horrida group	LC
*Acropora millepora* (Ehrenberg, 1834)	AY026449.1	[60]	aspera group	LNT
*Acropora monticulosa* (Brüggemann, 1879)	EF206471.1	[80]	humilis group	NT
*Acropora multiacuta* (Nemenzo, 1967)	EF206547.1	[80]	humilis group	V
*Acropora nasuta* (Dana, 1846)	AY026450.1	[60]	nasuta group	NT
*Acropora intermedia* (Dana, 1846)	AY026451.1	[60]	robusta group	LC
*Acropora palmata* (Lamarck, 1816)	AF505257.1	[73]	cervicornis group	CE
*Acropora papillare* (Latypov, 1992)	EU918211.1	[62]	aspera group	V
*Acropora pichoni* (Wallace, 1999)	EU918236.1	[62]	elegans group	NT
*Acropora prolifera* (Lamarck, 1816)	AF507266.1	[73]	cervicornis group	NOT ASSESSED
*Acropora pruinosa* (Brook, 1893)	AB638782.1	[82]	cf. divaricata group	DD
*Acropora pulchra* (Brook, 1891)	EU918230.1	[62]	aspera group	LC
*Acropora retusa* (Dana, 1846)	EF206537.1	[80]	humilis group	V
*Acropora robusta* (Dana, 1846)	FJ8990765	[77]	robusta group	LC
*Acropora rongelapensis* (Richards and Wallace, 2004)	EU918210.1	[62]	cf. loripes group	DD
*Acropora rotumana cf.* (Richards, Wallace, Miller, 2010)	FJ899069.1	[77]	cf. robusta group	NOT ASSESSED
*Acropora samoensis* (Brook, 1891)	AY364093.1	[80]	humilis group	LC
*Acropora sarmentosa* (Brook, 1892)	AY026455.1	[60]	florida group	LC
*Acropora selago* (Studer, 1878)	selago 1_25IF	[78]	selago group	NT
*Acropora solitaryensis* (Veron and Wallace, 1984)	AB638798.1	[82]	divaricata group	V
*Acropora spathulata* (Brook, 1891)	EU918242.1	[62]	aspera group	LC
*Acropora speciosa* (Quelch, 1886)	EU918244.1	[62]	loripes group	V
*Acropora spicifera* (Dana, 1846)	AY083881.1	[61]	aspera group	V
*Acropora tenella* (Brook, 1892)	EU918239.1	[62]	elegans group	V
*Acropora tenuis* (Dana, 1846)	AY026457.1	[60]	selago group	NT
*Acropora tortuosa* (Dana, 1846)	EU918238.1	[62]	horrida group	LC
*Acropora valenciennesi* (Milne Edwards and Haime, 1860)	val2_33IF	[78]	muricata group	LC
*Acropora valida* (Dana, 1846)	EU918235.1	[62]	nasuta group	LC
*Acropora vaughani* (Wells, 1954)	EU918224.1	[62]	horrida group	V
*Acropora walindii* (Wallace, 1999)	EU918234.1	[62]	elegans group	V
*Acropora yongei* (Veron and Wallace, 1984)	youngei2_15IF	[78]	selago group	LC
*Isopora cuneata* (Dana, 1846)	AY026429.1	[60]	Genus *Isopora* (included as outgroup)	V

The evolutionary history was inferred by using the Maximum Likelihood method based on the Kimura 2-parameter model [83] plus a discrete Gamma distribution was used to model evolutionary rate differences among sites (5 categories (+G, parameter = 0.9062)). The tree with the highest log likelihood (−2,744.0067) chosen by Bayesian Information Criterion is shown. The bootstap support values are shown next to the branches based on 1,000 replicates. The tree is drawn to scale, and branch lengths were measured as the number of substitutions per site. *Isopora cuneata* is included as the outgroup. 

## 5. Indices Providing Information about Climate Change Impacts on Aspects of PD

### 5.1. PD and Probabilities of Extinction — “Expected PD” Calculations

**Figure 2:**
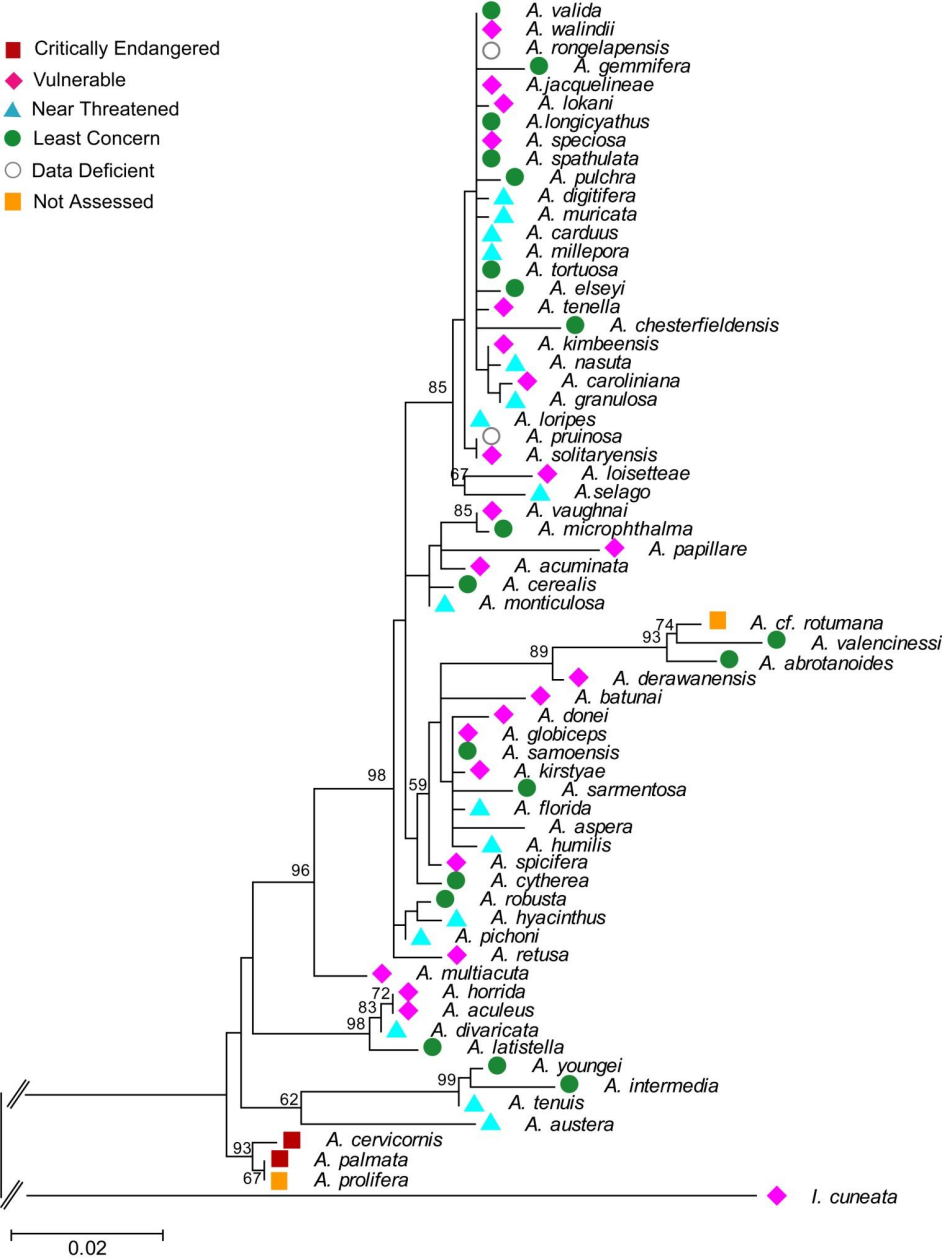
*Acropora* phylogeny inferred by Maximum Likelihood analysis with bootstrap support values indicated next to branches. The IUCN categories are provided for each species are colour coded (see inset to figure).

**Figure 3:**
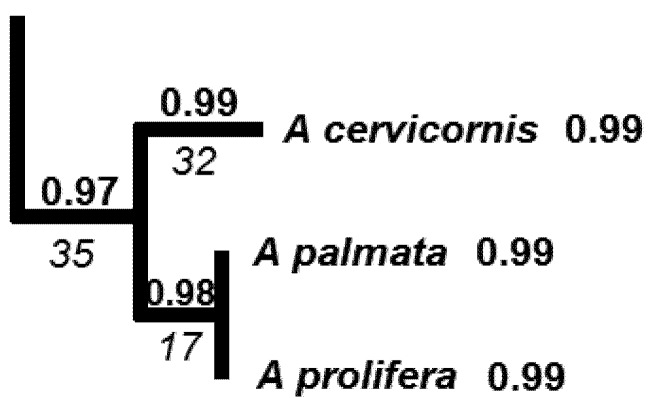
Subset of the larger tree (Figure 2) showing the three *Acropora* species found in the Caribbean. Relevant branch lengths are shown below branch, in italics. Bold numbers indicate probabilities of extinction, with probability for a deeper branch equal to product of probabilities of descendants.

In our brief review, we cited examples where species threats or extinctions were dispersed on the phylogenetic tree, so that consequent loss of PD was relatively small, for the given level of species loss. Based on our estimated phylogeny for the *Acropora* corals (Figure 2), we can conclude that we have a similar case of phylogenetically dispersed threats. However, the *Acropora* phylogeny suggests the need to move beyond the standard summary of PD loss over whole trees. For *Acropora*, a finer phylogenetic scale is of interest, because some parts of the tree show clumped threats. In one part of the tree (Figure 3), the three *Acropora* species found in the Caribbean form a monophyletic group, and two fall into the critically endangered category, while the third is not yet assessed (Figure 2). 

How do we best measure the potential impact on PD, including the loss of deeper branches, given the clumped impacts in this part of the tree? Here, we will consider indices that convert the IUCN threat categories (Table 1) to extinction probabilities. Higher threat categories are assigned higher probabilities of extinction [65,84]. Mooers *et al.* [65] discussed several possible transformations of category to extinction probability, and analyzed the sensitivity of conservation priorities to choice of transformation. Here, we will conservatively adopt the “pessimistic” transformation to extinction probabilities [65] (Table 1). 

One existing priority-setting approach based on extinction probabilities is the EDGE program (“evolutionarily distinct and globally endangered”) [84]. EDGE takes phylogeny into account in its calculations of priority scores for threatened species. A given species gains a credit, or partial contribution, from a given ancestral branch equal to 1/*n*, where *n* is the number of descendants of that branch. The total of these credits over all ancestral branches is then multiplied by the estimated extinction probability for the species for a final score. Species with higher scores receive higher conservation priority—they have ancestral branches with relatively few other descendants, and have highly threatened status.

The EDGE programme has promoted the practical use of phylogeny in conservation priority setting. However, it has been noted [85] that the method could be improved by incorporating existing PD‑based methods that incorporate probabilities (“expected PD”), as described by [86]. A key advantage over conventional EDGE calculations is that *complementarity* among species is accounted for effectively.

Here, the complementarity value of a given species reflects the degree to which related species do not already ensure the persistence of shared branches. A phylogenetically distinctive species that is only moderately threatened nevertheless may be given high conservation priority because it has high complementarity—there are no closely related secure species to ensure the persistence of its ancestral branches. The importance of complementarity in “expected PD” calculations has been illustrated for forest songbirds in the genus *Myadestes* [85] where the “Expected PD calculations would justify a relatively high conservation priority for *Myadestes obscures*, reflecting not only its increased extinction risk but also its elevated importance, given the expected loss of sister species, in representing the unique PD of this genus”.

**Figure 4:**
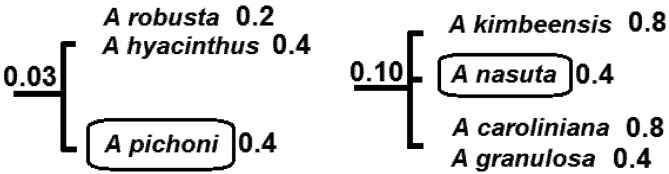
Two subsets of the larger phylogenetic tree (Figure 2). Two species that are currently near-threatened, *A. nasuta* and *A. pichoni*, are competing for conservation priority. Numbers indicate probabilities of extinction, with probability for a deeper branch equal to product of probabilities of descendants. *A. pichoni* has relatively secure sister species, while *A. nasuta* does not. Priority for *A. nasuta* would imply greater gain in expected PD.

We will illustrate this approach by examining two parts of the phylogeny of Figure 2, re-drawn in Figure 4. Suppose two species that are currently near-threatened, *A. nasuta* and *A. pichoni*, are competing for conservation priority. In each case, we will focus on the protecting the left-most branch shown in Figure 4 (the more ancestral branches to these are assumed secure, Figure 2). If we apply standard EDGE methods, *A. pichoni* would gain a large credit for its left-most ancestral branch (1/3, given the 3 descendants of that branch). *A. nasuta* has smaller credit for its left-most branch (1/4, given 4 descendants). The EDGE score then would be produced by multiplying these credit values by the estimated probability of extinction of the species. Here, near-threatened species, *A. nasuta* and *A. pichoni*, have the same probability, and *A. pichoni* gains EDGE priority, because it has greater credit for its ancestral branch (Figure 4). 

In contrast, expected PD calculations take into account the degree of threat to fellow descendants of any ancestral branch. For *A nasuta*, two of these species are vulnerable, with probabilities of extinction of 0.8 (Figure 2). The total probability of loss of the deep branch is 0.10. For *A. pichoni*, the sister species are quite secure, including one species of least concern (Figure 2 and Figure 4). The total probability of loss of the deep branch in this case is a much smaller value of 0.03. Therefore priority for *A. nasuta* would result in much greater gain in the expected persistence of phylogenetic diversity. In contrast, *A. pichoni* has relatively secure sister species, and so its ancestral PD is already well protected. It can be assigned lower priority. This example illustrates how the EDGE methods provide weaker priority setting when the objective is to maximize persistence of phylogenetic diversity.

With these lessons in mind, we now consider the Caribbean species (Figure 3). Two of the species, *A. cervicornis* and *A. palmata*, are critically endangered, and the third species, *A. prolifera*, is “not assessed”. For purposes of this example, we will consider a future scenario in which *A. palmata* is also designated critically endangered. The probability of loss of any terminal branch for critically endangered species is 0.99, based on the “pessimistic case” [65] for the conversion of the IUCN categories to extinction probabilities (Table 1). The probability of loss of the longer deep branch of length 35 (Figure 3) can be estimated as 0.99 × 0.99 × 0.99 = 0.97, assuming independent probabilities of extinction. This is the standard assumption for EDGE and expected PD methods. However, we note that the co-distribution of these 3 species in the heavily affected Caribbean region may mean that the probability of loss of the deeper branch is even higher. 

In this example, we can use expected PD calculations to derive an expected PD loss. This is the sum of each branch length times its corresponding probability of loss. The total expected PD loss is 82.29 branch length units = 32(0.99) + 17(0.98) + 35(0.97). We can see that the contribution of the deeper branch (0.97 times length of 34 = 33.0) to this expected PD loss is a high proportion of the total expected loss of 82.29. This calculation provides one simple index of the expected PD loss for phylogenetically-clumped impacts in a portion of a phylogenetic tree. 

A more informative index in practice would reflect the *changes* in expected PD loss that could be achieved under a particular candidate conservation action, for a given species or set of species. We extend the basic formulae [85,87], following the approach of [86,88], where the expected-PD change value for a set of species, S, is given by:

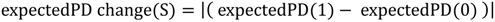


ExpectedPD(0) is the expected PD value, with extinction probabilities from current IUCN categories), and expectedPD(1) is the value when the probabilities are converted to a smaller value as a result of a conservation action. We will calculate an expected PD change value for the set of three Caribbean species (Figure 3). Here, expectedPD(0) is the expected PD calculation for this part of the tree, with extinction probabilities from current IUCN categories of 0.99 for each species. The expectedPD(1) is the value when the probabilities are converted to a smaller value—e.g., an extinction probability for the “near-threatened category (0.4). Because the change value reflects a change in expected PD as a consequence of changes in extinction probability for just these species, we do not have to calculate the expected PD for the entire tree.

This index reflects more than just the consequence of individual-species PD losses and can be applied to provide a score for conservation action for a region, as illustrated here for the Caribbean *Acropora*. When we assume that conservation action can change the extinction probabilities to those for the near-threatened category (0.4; Table 1), we have:

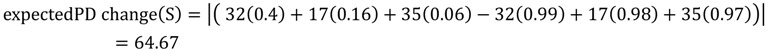


The improvement in expected PD, of 64.67 branch length units, is large compared to the original expected PD loss of 82.29. This improvement occurs even with an outcome where the species are still near-threatened. 

### 5.2. Phylogenetic Risk Analysis

**Figure 5:**
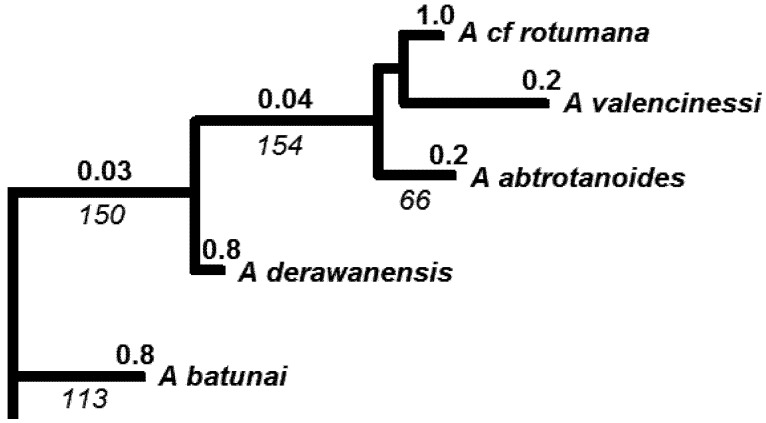
Subset of the larger phylogenetic tree (Figure 2). Relevant branch lengths are shown below branch, in italics. Estimated probabilities of extinction (following Table 1) for branches and for species are shown above the branches. Extinction probability for a deeper branch is the product of probabilities of descendants.

It has been argued that the focus of expected-PD assessments on finding good outcomes “on average” may not be the best way to avoid possible “worst-case” losses of PD [85] and that alternative risk aversion approaches for “phylogenetic risk analysis” are needed. Faith [85] presented a hypothetical example where the conservation option that best increased expected PD nevertheless implied a high probability of high PD loss, compared to other conservation options. An alternative conservation option would better avoid worst-case losses of PD, with only a small decrease in expected PD. We will explore this problem for a real phylogeny, again focusing on one portion of the larger corals tree (Figure 5).

Suppose we have some conservation budget that allows for either of two actions. *A. batunai* could be protected to the extent that its current probability of extinction of 0.8 could be reduced to a probability of extinction of 0.6. *A. abrotonoides* alternatively could be protected to the extent that its probability of extinction of 0.2 could be reduced to a probability of extinction of 0.0 (Figure 5). In order to maximize expected PD, we calculate the current expected PD and the change in expected PD for each alternative conservation action. Then we choose the conservation option with the greatest gain. The change in expected PD is the sum of all branch lengths, each multiplied by the change in the probability of extinction of the given branch. The conservation option focused on *A. batunai* would imply a change in expected PD of 22.6 (113 × 0.2; Figure 2). The alternative option focused on *A. abrotonoides* would imply a greater increase in expected PD (change value) of 24.1 (0.2 × 66 + 0.2 × 0.2 × 154 + .032 × 150 = 13.2 + 6.2 + 4.7; Figure 5). Maximizing expected PD therefore points to the option focused on *A. abrotonoides*. This choice, however, leaves open the high probability (0.8) that the relatively long branch leading to *A. batunai* will be lost. On average, the choice of *A. abrotonoides* may be a good one; however, choosing the best average outcome in this case is not sufficiently risk averse regarding the worst case loss of the relatively long branch of length 113 (Figure 5). If the *A. abrotonoides* conservation option is not selected, the probability of a PD loss as high as 113 is only 0.04. However, the possibility that maximizing an expected diversity outcome may not be sufficiently risk averse regarding possible worst case losses may be an important issue for conservation of corals PD; there are many cases where a threatened species is similar to *A. batunai* in being the sole descendent of a moderately long branch. 

No procedure for phylogenetic risk analysis was provided by [85]. However, expected PD calculations are flexible enough to be used to address this problem. While there seems to be no one formula that would provide a simple protocol for phylogenetic risk analyses, the best general guideline is to proceed as follows: nominate classes of “worst case” PD loss outcomes, and then assess the current probability of occurrence of these larger PD losses. Conservation options then can be assessed according to implied changes in these probabilities of worst-case losses, using the normal calculations for expected PD. 

### 5.3. Phylogenetic Spatial Homogeneity and Heterogeneity Analyses

Our review of studies of climate change impacts on PD pointed to a notable case where PD loss was relatively small, but there were dramatic projected changes in the geographic distribution of PD [38]. This study suggested that “the tree of life faces a trend towards homogenization across the continent” but these changes were not quantified. Measures of PD homogenization (or the opposite: PD spatial heterogeneity) would be useful for tracking changes over time and for making comparisons among taxonomic groups and among regions.

The term “biotic homogenization” refers to the increase in the biotic similarity of different localities in a region, and is now recognized as a major issue in biodiversity science (for review, see [89]). Typically, the focus is on the species level, but these indices suggest analogous measures of PD homogenization. Olden *et al.* [89] describe several species-level calculations: “taxonomic homogenization is calculated using species presence or absence data to examine the degree of similarity in community composition, and can be quantified using any one of a suite of similarity indices, diversity indices, cluster analyses or ordination approaches.” 

Olden *et al.* [89] noted that most homogeneity analyses use the Jaccard measure to calculate dissimilarity among localities. The Jaccard measure has a much-used phylogenetic counterpart based on PD [90]. The dissimilarity of two places/communities in this case reflects the count of the number of branches found (represented) in one place but not the other (see also [91]). This phylogenetic dissimilarity provides a simple PD-based spatial heterogeneity measure. Consider two localities or ecosystems, i and j. Let DPD(i,j) be the PD dissimilarity. We define the PD heterogeneity of the region, HPD1, as the average value of DPD(i,j) over all i and j. 

This measure may be useful for understanding changes in the PD distribution of corals as a result of possible climate change impacts. The geographic distribution of corals has been described based on 141 different eco-regions or ecosystems [92]. These can define “localities” for PD spatial heterogeneity assessments. Would loss of endangered coral species increase or decrease the global PD spatial heterogeneity? The three critically endangered species (Figure 2 and Figure 3) are only found in the Caribbean ecosystem types. This implies that a total branch length of 35 + 32 + 17 = 84 currently contributes to the PD dissimilarity of a Caribbean ecosystem to other global ecosystems. If the three Caribbean species were lost, the PD dissimilarity of a Caribbean ecosystem to other global ecosystems would appear to be smaller, contributing to a smaller HPD1. However, this extreme case where all *Acropora* are lost from the region has further implications. Those three species are the only *Acropora* species in the Caribbean. Consequently, not only those relatively short branches counted above but also the branch uniting all *Acropora* (Figure 2) is lost from the Caribbean ecosystem types. This loss of the deeper branch *increases* the PD dissimilarity of a Caribbean ecosystem to other global ecosystems, which have retained one or more *Acropora* species. Thus, the HPD1 measure would indicate an increase in spatial heterogeneity in this extreme case. In general, HDP1 could indicate lower heterogeneity for initial losses of species and branches and then greater heterogeneity as some ecosystems lose deeper branches while other ecosystems retain them. 

A possible limitation of HPD1 is that species and branches that are rare—for corals, found in few ecosystems—contribute to relatively few dissimilarities, among all *i* and *j*, and so will not have much influence on the overall value. Loss of rare PD therefore typically will not be reflected as a large change in HPD1. 

We suggest an alternative phylogenetic spatial heterogeneity measure that is more sensitive to changes in rare species, including the loss of rare PD. We follow other workers (e.g., [93] in creating a phylogenetic version of a general measure of diversity and evenness, developed by Hill [94]:

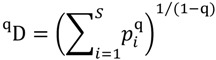


We follow the notation in Chao *et al.* [93], where ^q^D is the effective number of species, S is the number of species, and p_i_ is the proportional abundance of the ith species. The parameter q can be varied in order to give more or less weight to the most abundant features, defining a range of “Hill numbers”. When the ^q^D index value is large, the “effective number of species” is large, and we have greater heterogeneity.

Chao *et al.* [93] extended this fundamental measure of diversity and evenness to PD:

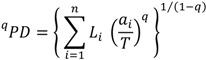


L_i_ is the length of branch i from the phylogenetic tree, and a_i_ is the total abundance of all species descended from branch I (T is the total of all a_i_). When q is 0, the measure is equivalent to PD. Chao *et al.* [93] offered no general phylogenetic model as a rationale for this measure. However, the basic rationale for PD—branch lengths as counting features (Section 2)—provides a simple justification for this PD-based “Hill numbers” framework. It can be interpreted as equivalent to applying the standard species-level Hills measure, but with features (as indicated by PD) substituted for species. 

For Chao *et al.*, the abundance of a branch is the summed abundance over descendant species. Here, we generalize this measure to cover the case where “abundance” reflects geographic abundance—that is, some measure of geographic range or rarity. We will consider number of localities (or ecosystems in the case of corals) containing a given branch as the abundance measure for that branch. This differs from the Chao *et al.* formulation in that the abundance for a branch is not the sum but the union over the abundance of descendants. We then define:

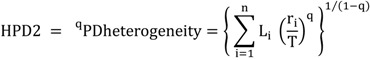

Our notation is the same as above, except that we use r_i_ to refer to the use of a range-type count as our abundance measure. When HPD2 is large, the effective amount of PD is large, and we have greater heterogeneity. We will explore the application of the measure for q equal to 1. The Hill number is undefined for q = 1, but, following [93,94], it is defined by a limit as q approaches 1, yielding:

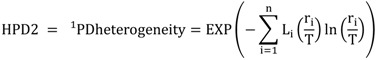

**Figure 6:**
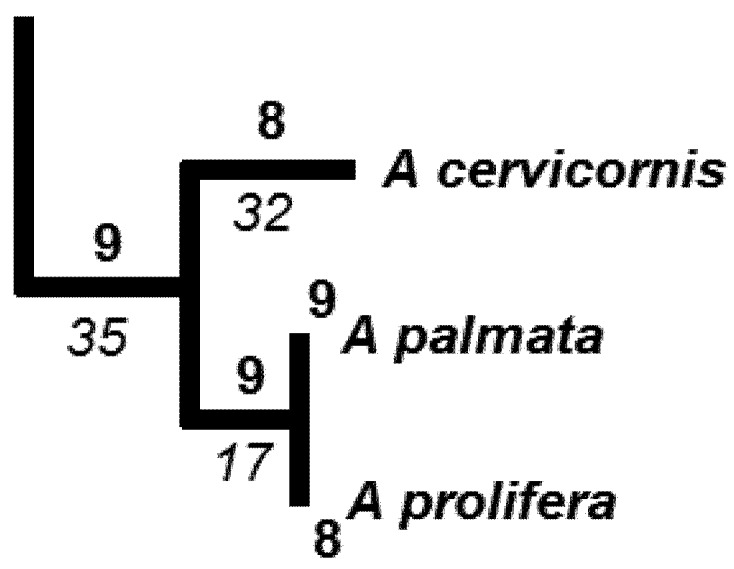
Subset of the larger tree (Figure 2) with the three *Acropora* species endemic to the Caribbean. Relevant branch lengths are shown below the branch, in italics. Numbers in bold at the top of the branches indicates the number of ecosystems in which the species or branch is represented (present) (according to [92]).

We now will apply this measure to the Caribbean species, using counts of the number of ecosystems containing a branch/species as the basis for abundance of that branch/species (Figure 6). For this example, we do not have information on the numbers of ecosystems for all other species and branches from the overall tree (Figure 2). However, we can illustrate properties of the method by considering an additional branch of length 32 that is found in a large number of ecosystems.

We begin by considering a case where the number of ecosystems for *A cervicornis* has dropped from 8 (Figure 6) to just 2 (scenario A in Table 3). The value of HPD2 is calculated as follows:

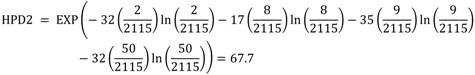


Now we compare this value of HPD2 to the value obtained for scenarios B and C (Table 3). In scenario B, *A cervicornis* becomes even rarer (present in only 1 ecosystem). HPD2 goes down, reflecting lower spatial PD heterogeneity. In contrast, in scenario C, the common species, at the end of a branch also of length 32, is now absent from one of its 50 ecosystems. HPD2 goes up, reflecting higher spatial PD heterogeneity. Thus, these simple scenarios suggest that HPD2 provides a measure of phylogenetic spatial heterogeneity that is sensitive to reductions in abundance of rare species/branches. This captures an important aspect of geographic homogenization from climate change impacts, and may provide a basis for the monitoring of PD change that complements the conventional monitoring of PD losses. 

**Table 3:** Scenarios of species loss from coral ecosystems, based on the partial tree of Figure 6. Column L_i _has branch lengths, including a hypothetical branch of length 32 from another part of the tree. Columns A, B, and C correspond to 3 scenarios for number of ecosystems for each species/branch. The bottom row reports, for each scenario, the corresponding HPD2 value.

L_i_	A	B	C
32	2	1	2
17	8	8	8
35	9	9	9
HPD2	67.7	64.6	68.2

## 6. Discussion

In our Introduction, we argued that phylogenetic diversity can contribute in two ways to a more inclusive earth systems science. First, we argued that phylogenetic pattern is a good proxy for the “features” that result from evolutionary processes, and so is part of a “life support system” that provides both current and possible future benefits. This reflects a long-standing rationale for the conservation of diversity [17]. The PD measure and its extended calculations introduced here are specifically designed to capture these phylogenetically-based option values of biodiversity. This perspective contrasts with a recent review and commentary on the conservation of phylogenetic diversity [95], which failed to recognize PD as a measure of feature diversity and associated option values. The result was an incoherent framework, with no clear rationale for the conservation of phylogenetic diversity and little basis for distinguishing among the large number of existing phylogenetic indices.

We noted that our focus on PD and option values complements standard systems approaches that largely focus on biodiversity links to ecosystem functions. Corals conservation highlights these complementary perspectives. Here, addressing the conservation of option values implied a focus on the entire phylogeny for that group (or sometimes hotspots of clumped threats within the phylogenetic tree). In contrast, a functioning coral ecosystem in a given place will be concerned with the subset of local taxa. This defines a portion of the larger phylogeny. Indeed, the most effective measure of biodiversity for assessments of ecosystem functions may be PD applied within ecosystems (see e.g., [96]). We have not addressed within-ecosystem PD analyses in this paper, but see the integration of whole-tree PD analyses and within-ecosystem PD analyses as a key challenge for an inclusive systems approach to sustainability.

This links to our second argument, that indices of change in phylogenetic diversity should assist in planning and decision-making for sustainability [97]. We see phylogenetic diversity conservation as contributing to a larger systems approach to sustainability (“regional sustainability analysis”, [21]) that investigates how we can balance different needs of society. These efforts will be progressed by efficient algorithms that can calculate best-possible changes in expected PD for large phylogenies, and integrated with human-environment factors, including conservation opportunity costs [98]. 

Our study has echoed some common themes from systems science, and perhaps has neglected some other important themes. On the one hand, we show that core systems ideas relating to tipping points and risk analysis extend to “evosystems”. On the other hand, our study did not explore the various uncertainties associated with these assessments. Naturally, phylogenetic diversity itself is recognition of inherent uncertainties regarding which species and which features will provide uses and benefits for future generations. However, use of phylogenetic pattern introduces other uncertainties. For example, as demonstrated here for *Acropora*, molecular data were not available for all species. Consequently, the species tree was based on a subset of the entire *Acropora* diversity. While the positioning of clades in the current phylogenetic topology is consistent with other published phylogenies [60,62], the inferred phylogeny is likely to be refined when additional species are included. PD assessments must take these uncertainties into account [99,100,101]. We hope that an inclusive systems approach, and availability of quantitative indices for phylogenetic diversity assessment, will promote new studies that overcome some of these uncertainties.
